# Enhanced RAD21 cohesin expression confers poor prognosis in BRCA2 and BRCAX, but not BRCA1 familial breast cancers

**DOI:** 10.1186/bcr3176

**Published:** 2012-04-26

**Authors:** Max Yan, Huiling Xu, Nic Waddell, Kristy Shield-Artin, Izhak Haviv, kConFab authors, Michael J McKay, Stephen B Fox

**Affiliations:** 1 Department of Anatomical Pathology, Prince of Wales Hospital, Barker Street, Randwick, 2031, Australia; 2 School of Medical Sciences, Faculty of Medicine, University of New South Wales, Kensington, 2052, Australia; 3 Research Division, Peter MacCallum Cancer Centre, St Andrews Place, East Melbourne, 3002, Australia; 4 Department of Pathology, Faculty of Medicine and Dental Sciences, The University of Melbourne, Grattan Street, Parkville, 3010, Australia; 5 Queensland Centre for Medical Genomics, Institute for Molecular Bioscience, University of Queensland, Brisbane, St Lucia, 4072, Australia; 6 Baker IDI Heart and Diabetes Institute, 75 Commercial Road, Prahran, 3004, Australia; 7 kConFab, Peter MacCallum Cancer Centre, St Andrew Place, East Melbourne, 3002, Australia; 8 North Coast Cancer Institute, Hunter St, Lismore, 2480, Australia; 9 Sydney Medical School, The University of Sydney, Camperdown, 2006, Australia; 10 Department of Pathology, Peter MacCallum Cancer Centre, St Andrews Place, East Melbourne, 3002, Australia

## Abstract

**Introduction:**

The *RAD21 *gene encodes a key component of the cohesin complex, which is essential for chromosome segregation, and together with BRCA1 and BRCA2, for high-fidelity DNA repair by homologous recombination. Although its expression correlates with early relapse and treatment resistance in sporadic breast cancers, it is unclear whether familial breast cancers behave in a similar manner.

**Methods:**

We performed an immunohistochemical analysis of RAD21 expression in a cohort of 94 familial breast cancers (28 BRCA1, 27 BRCA2, and 39 BRCAX) and correlated these data with genotype and clinicopathologic parameters, including survival. In these cancers, we also correlated RAD21 expression with genomic expression profiling and gene copy-number changes and miRNAs predicted to target RAD21.

**Results:**

No significant differences in nuclear RAD21 expression were observed between BRCA1 (12 (43%) of 28), BRCA2 (12 (44%) of 27), and BRCAX cancers (12 (33%) of 39 (*p *= 0.598). No correlation was found between RAD21 expression and grade, size, or lymph node, ER, or HER2 status (all *P *> 0.05). As for sporadic breast cancers, RAD21 expression correlated with shorter survival in grade 3 (*P *= 0.009) and but not in grade 1 (*P *= 0.065) or 2 cancers (*P *= 0.090). Expression of RAD21 correlated with poorer survival in patients treated with chemotherapy (*P *= 0.036) but not with hormonal therapy (*P *= 0.881). RAD21 expression correlated with shorter survival in BRCA2 (*P *= 0.006) and BRCAX (*P *= 0.008), but not BRCA1 cancers (*P *= 0.713). Changes in *RAD21 *mRNA were reflected by genomic changes in DNA copy number (*P *< 0.001) and by RAD21 protein expression, as assessed with immunohistochemistry (*P *= 0.047). High *RAD21 *expression was associated with genomic instability, as assessed by the total number of base pairs affected by genomic change (*P *= 0.048). Of 15 miRNAs predicted to target RAD21, mir-299-5p inversely correlated with RAD21 expression (*P *= 0.002).

**Conclusions:**

Potential use of RAD21 as a predictive and prognostic marker in familial breast cancers is hence feasible and may therefore take into account the patient's BRCA1/2 mutation status.

## Introduction

It is estimated that 5% to 10% of all breast cancers are attributable to inherited mutations, of which the two most important and highly penetrant are BRCA1 and BRCA2 [1]. Studies have demonstrated key differences in spontaneous BRCA-associated tumors [2,3]. BRCA1 cancers are more likely to show a basal phenotype, with 80% to 90% of BRCA1 cancers being negative for ER and HER2 and positive for basal cytokeratins [4,5]. BRCA1 cancers also have characteristic gene-expression and genomic profiles, and appear to be sensitive to DNA damage by cisplatinum. Although reports suggest that lobular carcinomas may be more frequent in BRCA2 carriers, no specific molecular phenotype has been described for BRCA2-associated tumors, which usually show a ductal, no-special-type morphology and ER positivity [6].

RAD21 is a component of the multiprotein complex cohesin, which is involved in maintaining alignment and cohesion of replicated "sister" chromatids. RAD21, together with SMC1, SMC3, and STAG1/2, forms a tripartite ring, which according to the "ring model," promotes sister chromatid cohesion (SCC) by encircling sister chromatids [7]. SCC not only is vital for correct chromosome segregation during mitosis and meiosis, but also plays an important role in the repair of double-stranded DNA breaks (DSBs). By promoting sister-chromatid alignment, cohesin allows BRCA1/2-mediated homologous recombination to occur between sister chromatids [8]. In addition to its role in maintaining sister-chromatid cohesion, evidence now suggests cohesins are involved in promoting and inhibiting gene transcription. In MCF-7 cell lines, genes cobound by cohesin and ER are preferentially regulated by estrogen [9]. Cohesin may also act as a negative regulator of gene expression, by physically blocking enhancer/promoter interaction [10].

Of the four proteins that compose the core cohesin complex, RAD21 has emerged as a key marker of tumor behavior. A meta-analysis of gene-expression data from clinical cancer specimens showed that increased *RAD21 *expression was a feature of poorly differentiated breast, ovarian, bladder, and lung cancers [11]. Gene-expression profiling of 31 breast cancer patients with supraclavicular lymph node metastasis revealed *RAD21 *as one of six genes that were differentially expressed between good- and poor-outcome groups [12]. Our previous study on sporadic breast cancers showed that RAD21 overexpression correlated with early relapse in high-grade breast cancers regardless of intrinsic tumor subtype [13]. We, and others, also showed that *RAD21 *knockdown confers *in vitro *resistance to DNA-damaging chemotherapeutic agents, which recapitulated the findings in our cohort of sporadic breast cancers [13,14].

Although RAD21 overexpression correlates with early relapse and treatment resistance in sporadic cancers, it is unclear whether familial breast cancers behave in a similar manner. This may be of particular relevance, as RAD21, BRCA1, and BRCA2 are all involved in DNA repair through homologous recombination; hence RAD21 overexpression in the absence of either BRCA1 or -2 may not necessarily confer the same predictive and prognostic implications as in sporadic cancers with intact BRCA1/2. We therefore performed an immunohistochemical analysis of RAD21 expression in a cohort of familial breast cancers. We also postulated that enhanced RAD21 expression may be associated with changes in both DNA copy number and reduced expression of microRNAs (miRNAs), and therefore correlated RAD21 expression with genomic changes and miRNAs predicted to target RAD21.

## Materials and methods

### Patients

Breast cancer specimens were collected from a previously characterized cohort of 139 female patients from the kConFab family breast cancer registry (Table 1) [15]. Classification of BRCA1, BRCA2, and BRCAX status was performed as described previously [15]. The flow of patients according to the REMARK guidelines (Additional file 1 Table S1) [16]. Of the 139 cases, 18 cases were excluded because of the lack of tissue available for array construction, and a further 27 cases were excluded because of the absence of tumor on the array stained for RAD21. The final cohort was composed of 94 cases, which included 28 BRCA1, 27 BRCA2, and 39 BRCAX cases. This study has ethics committee approval (Peter MacCallum Cancer Centre, 09/36). Consent to participate in the study and consent to publish were obtained by kConFab in accordance with its family-enrollment and data-collection guidelines [17]. Patients were followed up for a median period of 64.0 months (range, 0.4 to 299.0 months). During this time, 38 patients relapsed, and 33 died of breast cancer (deaths unrelated to breast cancer were censored). Relapse-free survival was defined as the time to first reappearance of tumor at any site after definitive treatment, whereas breast cancer-specific survival was defined as time from primary surgical excision to breast cancer-related death. All patients with HER2-positive tumors were diagnosed prior to 2000, and did not receive trastuzumab therapy.

**Table 1:** Clinical and tumor characteristics

	BRCA1 *n *(%)	BRCA2 *n *(%)	BRCAX *n *(%)	All familial *n *(%)
**Age**				

≤ 50 years	25 (89%)	15 (56%)	24 (61%)	64 (68%)

> 50 years	3 (11%)	12 (44%)	15 (39%)	30 (32%)

**Tumor size**				

< 20 mm	20 (71%)	13 (52%)	16 (47%)	49 (56%)

> 20 mm	8 (29%)	12 (48%)	18 (53%)	38 (44%)

Unknown	0	2	5	7

**Nodal status**				

Negative	26 (93%)	19 (70%)	25 (64%)	70 (74%)

Positive	2 (7%)	8 (30%)	14 (36%)	24 (26%)

Unknown	0	0	0	0

**Grade**				

I	0	1 (4%)	2 (6%)	3 (4%)

II	2 (9%)	10 (44%)	8 (24%)	20 (25%)

III	21 (93%)	12 (52%)	23 (70%)	56 (71%)

Unknown	5	4	6	15

**ERα**				

Negative	23 (85%)	5 (21%)	11 (32%)	39 (46%)

Positive	4 (15%)	19 (79%)	23 (68%)	46 (54%)

Unknown	1	3	5	9

**PgR**				

Negative	23 (85%)	9 (38%)	16 (47%)	48 (56%)

Positive	4 (15%)	15 (62%)	18 (53%)	37 (44%)

Unknown	1	3	5	9

**HER2 status**				

Negative	26 (100%)	22 (100%)	28 (85%)	76 (94%)

Positive	0	0	5 (15%)	5 (6%)

Unknown	2	5	6	13

**Endocrine therapy**				

Not given	22 (96%)	16 (76%)	21 (64%)	59 (77%)

Given	1 (4%)	5 (24%)	12 (36%)	18 (23%)

Unknown	5	6	6	17

**Chemotherapy**				

Not given	8 (30%)	15 (60%)	13 (39%)	16 (42%)

Given	19 (70%)	10 (40%)	20 (61%)	49 (58%)

Unknown	1	2	6	9

*n *= 94.

### Immunohistochemistry

Tumor-tissue microarrays (1-mm cores), with a twofold redundancy, were prepared from archival formalin-fixed, paraffin-embedded tissue blocks. RAD21 staining was performed as previously described by using a rabbit polyclonal anti-RAD21 antibody (1:200; Abcam, Cambridge, UK) [13]. Nuclear RAD21 expression was assessed for intensity (0, no staining; 1, weak; 2, moderate; 3, strong) and the percentage of positive cells (0, 0; 1, < 10%; 2, 10% to 50%; 3, 51% to 80%; 4, > 80% positive cells). The scores for intensity and percentage were added, and a cut-off of 6, the median, was used to define two approximately equal sized groups of patients (with low and high RAD21 expression) for subsequent statistical analyses [13].

HER2 chromogenic *in situ *hybridization (CISH) and immunoperoxidase staining for ERα, PgR, HER2, CK5/6, and EGFR were performed for all tumors. By using stratification of intrinsic phenotypes based on Nielsen *et al. *[18], we placed tumors into luminal (ER positive, HER2 negative, cytokeratin (CK) 5/6 negative or positive), basal (HER2 and ER negative; CK5/6 positive), HER2 (HER2 positive, ER and CK5/6 negative or positive), and null/negative (HER2, ER, and CK5/6 negative). For HER2, EGFR, and CK5/6, the cut-offs were derived from Neilsen *et al. *[18]. An Allred score of > 2/8 was considered positive for ERα [19].

### Gene-expression and copy-number analysis of *RAD21 *

Gene-expression and copy-number variation (CNV) data from a cohort of familial breast cancers were derived from a previous study, by using the Illumina Human-6 BeadArray (Illumina, San Diego, CA, USA) and the CNV370 SNP array (Illumina), respectively [20]. In this study, the familial tumors were classified into one of the breast tumor subtypes: basal-like, luminal A, luminal B, HER2-positive, and normal-like. These data were used to determine the expression of RAD21 in 75 (19 BRCA1, 30 BRCA2, 25 BRCAX, one unknown) familial breast cancers. Of these 75 cases, 34 had matching CNV data available (11 BRCA1, nine BRCA2, and 14 BRCAX), and 18 had matching protein-expression data as assessed by immunohistochemistry (seven BRCA1, eight BRCA2, three BRCAX). Copy number was determined by using SNP-CGH data for 34 tumors. All data were imported and visualized in Beadstudio v3.2. The logR ratio was used to perform frequency plots of genomic gain or loss by using CGH explorer [21]. R was used to perform SOMATICS [22] to identify regions containing genomic aberrations. The copy number of *RAD21 *in each tumor was inferred from the average logR value of 22 single-nucleotide polymorphisms (SNPs), which were within the RAD21 intron (*n *= 2) or in sequences flanking the gene (*n *= 20). All these data are available on GEO (Accession Number GSE19177).

### microRNA and gene-expression data mining

Matching gene-expression and miRNA profiles of 215 sporadic breast cancer specimens were obtained by mining a microarray dataset described by Buffa *et al. *[23], in GEO (Accession number GSE22220), samples BCmicroRNA 1 to 110). Normalized log2 signal intensities were obtained after background subtraction and quantile normalization, as previously described by Buffa *et al. *[23]. This signal intensity for *RAD21 *was used for survival analysis, as outlined subsequently.

### microRNA microarray

miRNA expression was assessed for 11 BRCA1 basal breast cancers and 13 normal breast specimens via microarrays. For each sample, 250 ng of total RNA was labeled and hybridized on Human v2 MicroRNA Expression BeadChips (Illumina). The BeadChips were scanned with the Illumina iScan Reader [24]. Data were imported into GenomeStudio (Illumina), from which raw data with background subtraction were exported to the PARTEK Genomics Suite (St. Louis, MO, USA) for further analysis. Raw probe intensities were shifted, such that the minimum probe intensity for each sample was equal to 1. All values were transformed by taking logs (base 2), followed by quantile normalization [25,26]. Probe mapping for Illumina MicroRNA Expression v2 BeadChips was based on miRBase v.12.0 [27].

### Statistical analysis

Correlations were evaluated by using the Mann-Whitney *U *or χ^2 ^tests where appropriate. Kaplan-Meier survival curves were calculated for breast cancer-specific death and were compared with the log rank test. The Cox proportional hazard regression model was used to identify independent prognostic factors for breast cancer-specific survival. Analyses were performed with SPSS 16.0 software (SPSS Inc., 233 South Wacker Drive, Chicago, IL, USA). A two-tailed *P *value test was used in all analyses, and a *P *value of less than 0.05 was considered statistically significant.

## Results

### RAD21 protein expression in familial breast cancers, their relation with intrinsic subtypes and clinicopathologic parameters

**Figure 1:**
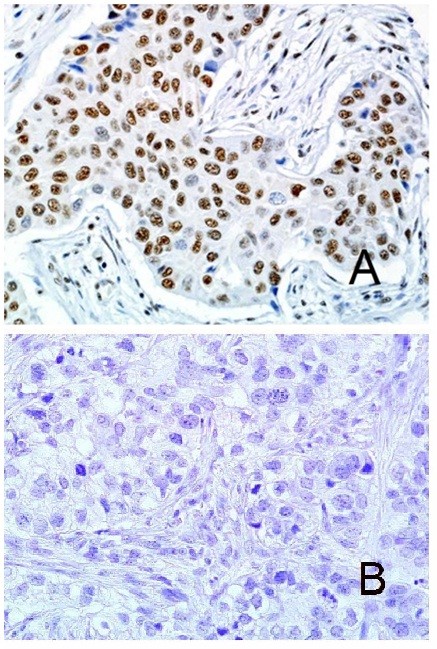
**Expression in invasive carcinoma**. **(A) **High RAD21 nuclear expression in invasive carcinoma. **(B) **Absent RAD21 expression in invasive carcinoma.

The median age of the cohort was 45.6 years. No difference was found in breast cancer-specific survival when stratified by age, by using a cut-off of 40 years (*P *= 0.442). The tumors in our cohort showed either absent, low, or high RAD21 expression (as defined earlier; Figure 1). With direct comparison of individual expression scores or a median cut-off score, no significant differences in nuclear RAD21 expression were observed between BRCA1 (12 (43%) of 28), BRCA2 (12 (44%) of 27), and BRCAX cancers (12 (33%) of 39) (*P *= 0.598) (Additional file 2 Table S2a). Similarly, no differences in Rad21 expression were seen within the intrinsic breast cancer subtypes (luminal, 17 (42%) of 41; basal, 14 (44%) of 32; HER2, two (50%) of four; and null, one (20%) of five; *P *= 0.768) (Additional file 2 Table S2b). No correlation was seen between RAD21 expression and tumor grade, size, lymph node status, or ER or HER2 status (all *P *> 0.05, Table 2).

**Table 2:** Correlation of RAD21 expression with clinicopathologic features (*n *= 94)

	RAD21 negative	RAD21 positive	*P *value
**Age**			
	
≤ 50 years	40 (70%)	24 (65%)	0.589
	
> 50 years	17 (30%)	13 (35%)	

**Tumor size**			
	
< 20 mm	29 (55%)	20 (59%)	0.706
	
> 20 mm	24 (45%)	14 (41%)	

**Nodal status**			
	
Negative	42 (74%)	28 (76%)	0.829
	
Positive	15 (26%)	9 (24%)	

**Grade**			
	
I	2 (5%)	1 (3%)	0.128
	
II	7 (16%)	13 (36%)	
	
III	34 (79%)	22 (61%)	

**ERα**			
	
Negative	22 (44%)	17 (49%)	0.677
	
Positive	28 (56%)	18 (51%)	

**HER2 status**			
	
Negative	46 (96%)	30 (91%)	0.366
	
Positive	2 (4%)	3 (9%)	

**Relapse**			
	
Negative	44 (79%)	18 (51%)	0.007
	
Positive	12 (21%)	17 (49%)	

**BCSS**			
	
Negative	45 (80%)	19 (54%)	0.006
	
Positive	11 (20%)	16 (46%)	

BCSS, Breast cancer-specific survival.

### RAD21 expression and survival in familial breast cancers

**Figure 2:**
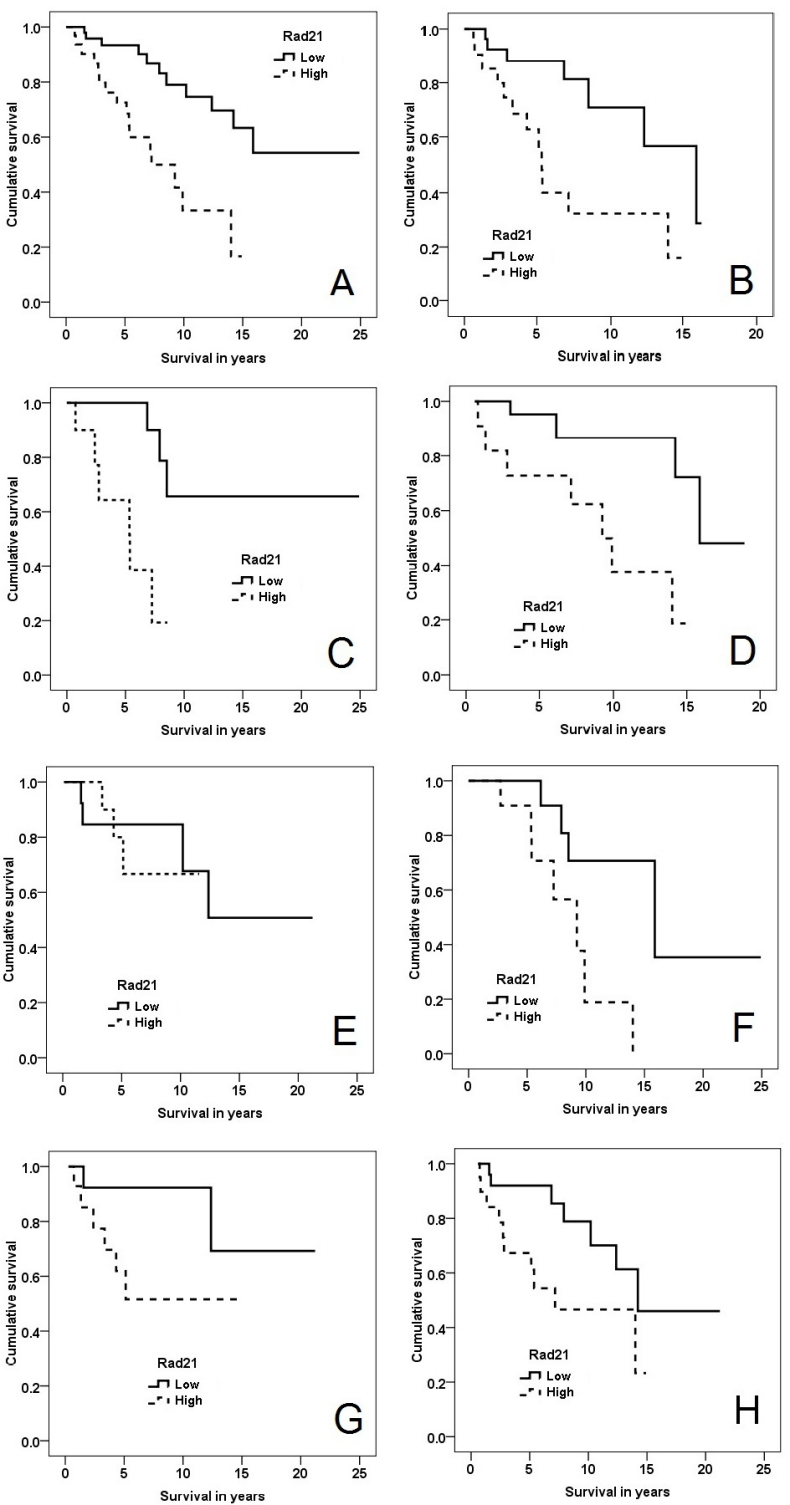
**Kaplan-Meier curves, breast cancer-specific overall survival stratified by Rad21 expression**. **(A) **All familial cancers (*P *= 0.001). **(B) **Grade 3 familial cancers (*P *= 0.009). **(C) **BRCA2 cancers (*P *= 0.006). **(D) **BRCAX cancers (*P *= 0.008). **(E) **BRCA1 cancers (*P *= 0.071). **(F) **Familial luminal cancers (*P *= 0.010). **(G) **Familial basal cancers (*P *= 0.075). **(H) **Familial cancers treated with adjuvant chemotherapy (*P *= 0.036).

A significant correlation was noted between high-RAD21 expression and shorter relapse-free survival (*P *= 0.038) and breast cancer-specific survival (*P *= 0.001, Figure 2A) across the entire familial cancer group. Correlation with breast cancer-specific survival was confirmed in a multivariate analysis (including ER, HER2, tumor grade, size, lymph-node status), with RAD21 as a continuous score out of seven (homologous recombination (HR) = 1.66; *P *= 0.003; 95% CI, 1.19 to 2.32; Table 3). Similar to our previous findings in sporadic breast cancers [13], high RAD21 expression correlated with poorer relapse-free survival (*P *= 0.008) and breast cancer-specific survival for grade 3 familial breast cancers (*P *= 0.009, Figure 2B) [13], but not for grade 1 and 2 cancers (*P *= 0.065 and 0.090, respectively).

**Table 3:** Cox regression model: factors influencing breast cancer-specific survival in familial breast cancers

	*P *value	Hazard ratio	95% Confidence interval
**RAD21 (score out of 7)**	0.003	1.66	1.19-2.32

**ER**	0.088	0.33	0.09-1.18

**HER2**	0.088	2.24	0.51-9.97

**Grade**	0.030	5.66	1.18-27.22

**Size**	0.691	0.99	0.97-1.02

**Lymph-node status**	0.003	6.05	1.82-20.15

Correlation of RAD21 expression with breast cancer-specific survival was further assessed for BRCA1, BRCA2, and BRCAX cancers. Interestingly, high RAD21 expression correlated with poorer survival in BRCA2 (*P *= 0.006) (Figure 2C) and BRCAX cancers (*P *= 0.008) (Figure 2D), but not in BRCA1 cancers (*P *= 0.71) (Figure 2E). When the intrinsic subtypes were individually analyzed, high RAD21 expression correlated with worse survival in luminal breast cancers (*P *= 0.010) (Figure 2F). A similar divergence of the survival curves was observed in basal cancers, although this was not statistically significant (*P *= 0.075) (Figure 2G). Insufficient numbers of HER2 and null-type cancers were available for survival analyses to be performed (*n *= 2 and 3, respectively).

High RAD21 expression correlated with shorter survival for patients treated with adjuvant chemotherapy (*P *= 0.036) (Figure 2H). Expression of RAD21 did not correlate with survival in patients receiving hormonal therapy (*p *= 0.88).

### Validation of RAD21 expression as a prognostic marker in the cohort of sporadic cancers from Buffa *et al*

**Figure 3:**
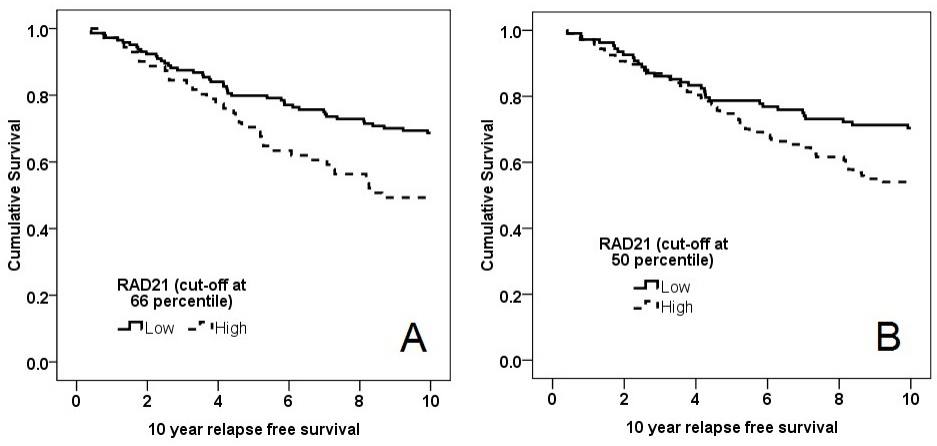
**The 10-year relapse-free survival of 215 patients from Buffa *et al. ***[23], **stratified by *RAD21 *gene expression**. **(A) **Cut-off at 66^th ^percentile (that is, top third), and **(B) **cut-off at 50^th ^percentile (that is, top half).

The correlation of *RAD21 *gene expression to 10-year relapse-free survival was explored in a validation cohort of 215 breast cancer patients, with tumors previously characterized on the Illumina Human RefSeq-8 microarray, by Buffa *et al. *[23]. Kaplan-Meier curves were charted after stratifying the cohort into two groups, with high *RAD21 *expression being defined at the 66^th ^percentile (that is, top third of tumors). The same analysis was repeated with the cut-off set at the 50^th ^percentile. High *RAD21 *expression correlated with poorer survival at both cut-offs (*P *= 0.007 and *P *= 0.024, respectively; Figure 3). At a cut-off at the 66^th ^percentile, *RAD21 *was an independent indicator of 10-year relapse-free survival in a multivariate analysis including ER status, lymph node status, grade, size, and age (HR = 1.62; *P *= 0.046; 95% CI, 1.08 to 2.61).

### Gene expression correlates with relative copy number and protein expression of RAD21 in familial breast cancers

**Figure 4:**
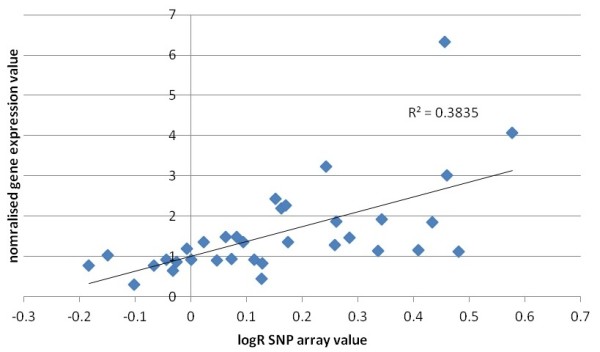
**Correlation between *RAD21 *gene copy-number changes as assessed by the average logR array value of 24 single-nucleotide polymorphisms (SNPs)**. These were located in RAD21 or within the flanking region of the gene and normalized expression of RAD21 in 34 breast cancers.

The cohort of familial tumors was previously analyzed on the basis of gene-expression and copy-number analysis [20]. No significant difference was found in *RAD21 *gene expression between BRCA1, BRCA2, and BRCAX cancers (*P *= 0.170, Kruskal-Wallis test). Similarly, no significant difference was noted in *RAD21 *copy number among the BRCA subtypes (*P *= 0.141). Similar to our findings in sporadic breast cancers, no difference was seen in *RAD21 *copy number between basal-like and luminal cancers (*P *= 0.749). The 34 tumors with both gene-expression and copy-number data showed a significant correlation between *RAD21 *expression and estimated copy number (*r *= 0.619; *P *< 0.001) (Figure 4). Five (15%) of the 34 tumors showed a copy-number gain (copy number of 3). Of these five tumors with copy number gain, there were four basal-like tumors and one luminal tumor.

**Figure 5:**
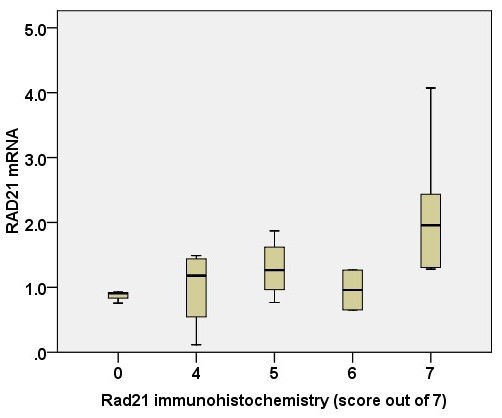
**Correlation of *RAD21 *gene expression with RAD21 immunohistochemistry (score out of 7)**. *r *= 0.475; *P *= 0.047.

For 18 tumors, *RAD21 *gene expression was matched with protein expression, as assessed by immunohistochemistry. This showed a significant correlation between *RAD21 *gene expression and RAD21 protein expression (of seven) (*r *= 0.475; *P *= 0.047) (Figure 5).

### *RAD21 *expression is linked with genomic instability in familial breast cancers

**Figure 6:**
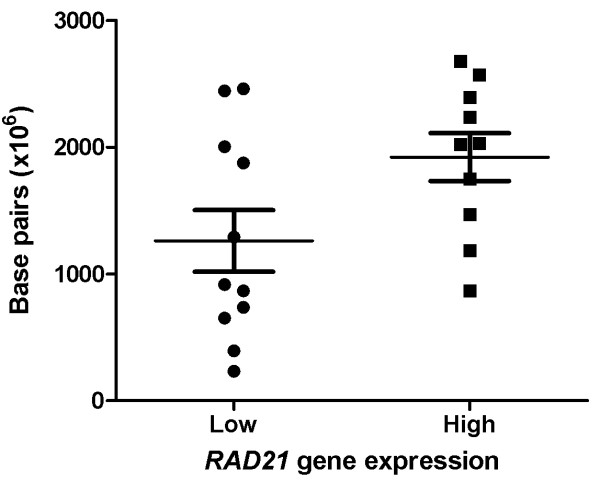
**Comparison of the total number of base pairs affected by genomic change between tumors with low (*n *= 11) and high (*n *= 11) *RAD21 *gene expression, *P *= 0.048**.

Thirty-four tumors were analyzed for genomic change by using SNP-CGH profiling, as previously described by Waddell *et al. *[20]. SOMATICS [22] was used to identify copy-number change and copy-neutral loss of heterozygosity. The total number of chromosomal aberrations and the total number of base pairs affected by genomic change were compared between high *RAD21 *(top third; *n *= 11) and low *RAD21 *(bottom third; *n *= 11) expressing tumors, as assessed by gene-expression analysis. Tumors with high *RAD21 *expression had a higher number of base pairs affected by genomic change (mean = 1.92 × 10^9^), compared with tumors with low *RAD21 *expression (mean = 1.26 × 10^9^; *P *= 0.048; Figure 6). Although no significant difference was found in the total number of chromosomal aberrations between high and low *RAD21-*expressing tumors (*P *= 0.660), the difference in the number of base pairs affected by genomic change suggests that *RAD21 *expression is linked with a higher level of genomic instability.

### mir-299-5p is predicted to target *RAD21 *and inversely correlates with *RAD21 *expression in sporadic breast cancers

A search of potential miRNAs that may target *RAD21 *was performed on MicroCosm Targets, version 5 [28]. The correlation between *RAD21 *gene expression and the expression of 15 miRNAs predicted to target *RAD21 *was interrogated by mining matching gene-expression and miRNA-array data from the cohort of Buffa *et al. *(108 tumors from miRNA arrays 1 to 110) [23]. Of the 15 miRNAs examined, mir-299-5p inversely correlated with RAD21 expression (Pearson *r *= -0.294; *P *= 0.002).

## Discussion

Although RAD21 expression is associated with a poor prognosis and treatment resistance in sporadic breast cancers, the role of RAD21 in familial breast cancers is unclear, as RAD21 expression in the absence of either functional BRCA1 or 2 may not necessarily confer the same predictive and prognostic implications as in sporadic cancers with intact BRCA1/2. We therefore performed the first analysis of RAD21 in a cohort of fully characterized familial breast cancers and investigated potential mechanisms that may mediate RAD21 levels, including DNA copy number and reduced expression of targeting microRNA.

Our findings in a cohort of familial breast cancers recapitulated our previous findings in sporadic breast cancers, with enhanced expression of RAD21 occurring in a subset of tumors regardless of grade, size, or intrinsic subtype [13]. Similarly, RAD21 expression conferred a poor prognosis in grade 3, but not in grade 1 or 2 cancers. The previously demonstrated correlation of RAD21 with survival was also confirmed in the cohort of Buffa *et al. *of sporadic cancers assessed with gene-expression arrays and in our cohort of familial breast cancers.

We found that RAD21 expression does not correlate with BRCA status. However, its expression associates with a poor prognosis in BRCA2 and BRCAX cancers. This finding is consistent with our previous findings in sporadic cancers [13]. Interestingly, RAD21 was not associated with a poor prognosis in BRCA1 cancers. How RAD21 expression influences the poor prognosis in BRCA2 and BRCAX but not in BRCA1 patients remains to be determined. This finding is likely to be explained by the final roles of RAD21 and BRCA1/2 in homologous recombination (HR). Like BRCA1 and BRCA2, cohesins are important regulators of genomic stability. Cohesins facilitate error-free repair of double-stranded breaks (DSBs) in DNA by HR, possibly by promoting the alignment and cohesion of sister chromatids [8]. Defects in HR result in DSB repair through the alternative, error-prone, nonhomologous end-joining and single strand-annealing pathways; this may lead to deletions, translocations, and chromosomal instability [29]. HR is particularly important in the repair of DNA damage caused by chemotherapeutic agents and radiotherapy. Although both BRCA1 and BRCA2 participate in HR, evidence from genetic studies suggests that BRCA1 functions upstream of BRCA2 [30]. Hence, in the setting of BRCA1 cancers in which DSB repair has already been compromised by deleterious BRCA1 mutations, overexpression of RAD21 may have no effect on tumor behavior. Furthermore, removal of the cohesin complex after DNA repair requires proteolytic cleavage of RAD21 by caspase 3 [31]. Activation and translocation of caspase 3 into the nucleus requires functioning wild-type BRCA1 and is inhibited by mutated BRCA1 [32]. Failure to cleave RAD21 due to BRCA1-dependent caspase 3 inhibition may explain loss of HR repair in BRCA1 but not in BRCA2 cancers.

Another possible explanation relates to the emerging role of cohesin as a key regulator of gene transcriptions [33,34]. RAD21 is implicated in mediating estrogen-regulated transcription through ERα in MCF7 breast cancer cells [9]. Hence RAD21 expression may influence a subset of gene expression through interplay with ERα, and thereby contribute to the poor prognosis in BRCA2 and BRCX patients. This notion is consistent with ER status in our cohort, in which 85% of BRCA1 tumors are ERα negative. Although RAD21 may be implicated in ER-regulated gene expression, functional genomic screens performed on estrogen-dependent cell lines have so far yielded conflicting results regarding the role of RAD21 in tamoxifen resistance. Silencing of *RAD21 *via sh-RNA suggests that RAD21 expression is associated with sensitivity to tamoxifen [35], whereas transduction by retroviral cDNA suggests that RAD21 expression is associated with resistance [36]. Neither of these findings appears to be reflected by our clinical cohorts, as Rad21 expression did not correlate with survival in either sporadic (*P *= 0.231) or familial (*P *= 0.881) breast cancers treated with hormone therapy.

Our previous study demonstrated that RAD21 expression is associated with chemotherapy resistance in cell lines and in a cohort of sporadic cancers [13]. Similarly, in our cohort of familial cancers receiving adjuvant chemotherapy, RAD21 expression correlated with a poorer prognosis. This is despite our finding that RAD21 does not correlate with survival in BRCA1 cancers and the higher use of chemotherapy in BRCA1 cancers. This further supports our argument that BRCA1 cancers behave differently from other cancers in response to RAD21 overexpression.

Although the function of RAD21 has been extensively investigated in the literature, relatively little is known regarding the regulation of its expression. In keeping with our previous findings in sporadic breast cancers [13], *RAD21 *gene expression correlated with copy number in our cohort of familial breast cancers. This is further supported by the positive correlation between *RAD21 *gene expression and protein expression, as assessed by immunohistochemistry in matched tumor samples. Increased *RAD21 *expression is associated with increased numbers of base pairs being affected by genomic change, which suggests that increased expression is linked to genomic instability. The effect of genomic changes on RAD21 expression appears to be independent of BRCA status and intrinsic tumor subtype. RAD21 upregulation may be an early event that occurs before tumor invasion. This is supported by the presence of increased RAD21 expression in 46% of the DCIS cases we previously examined [13]. In addition, interrogation of the cohort of Schuetz *et al. *[37] showed no difference in RAD21 expression between 14 microdissected matching invasive and *in situ *ductal carcinoma samples (*P *= 0.154).

**Figure 7:**
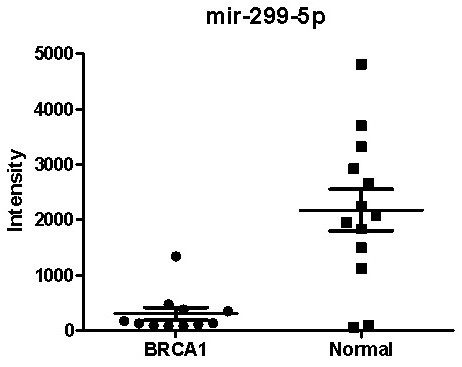
**Comparison of mir-299-5p expression between BRCA1 basal cancers (*n *= 11) and normal breast tissue (*n *= 13)**.

In addition to genomic changes, we postulated that other mechanisms are likely to control *RAD21 *expression, such as through targeting microRNAs. Of the 15 miRNAs predicted to target *RAD21*, mir-299-5p expression significantly inversely correlated with *RAD21 *expression. Reduced circulating mir-299-5p was previously demonstrated in the serum of breast cancer patients [38], compared with normal controls. Silencing of mir-299-5p has also been implicated in the pathogenesis of oral squamous cell carcinomas [39]. Limited data derived from breast cancer cell lines showed that mir-299-5p knockdown is associated with upregulation of osteopontin [40], a glycoprotein involved in invasion, metastasis, and resistance to radiotherapy and chemotherapeutic agents [41,42]. Aberrant regulation of *RAD21 *by mir-299-5p is also supported by a comparison of 11 BRCA1 basal and 13 normal breast samples, in which a sevenfold reduction in mir-299-5p was observed between BRCA1 cancers and normal breast tissue (*P *= 0.0002; Figure 7). Nevertheless, although mir-299-5p is clearly reduced in breast cancer, this appears appear to play a relatively minor role in regulating *RAD21 *expression (*r*^2 ^= 0.0864; *P *= 0.002) compared with genomic changes in copy number (*r*^2 ^= 0.286; *P *< 0.001).

In summary, our findings show that RAD21 expression is associated with a poorer prognosis in BRCA2 and BRCAX, but not in BRCA1 cancers. Because increased RAD21 expression may confer resistance to DNA-damaging agents, alternative treatment strategies may be useful in RAD21-positive BRCA2 and BRCAX cancers. This may include dosage intensification and the use of chemotherapeutic agents with alternative modes of action. The efficacy of poly-ADP ribose polymerase (PARP)-inhibitor therapy is dependent on defective HR repair [43,44]. In view of the role of RAD21 in increasing HR activity [33], tumor RAD21 status may be particularly relevant in patients being considered for PARP-inhibitor therapy.

## Conclusions

RAD21 expression in familial cancers is reflected by expression changes in copy-number expression. It is also inversely correlated with mir-299-5p, the expression of which is suppressed in breast cancers. Similar to our earlier findings in sporadic breast cancers, increased RAD21 expression in BRCA2 and BRCAX cancers confers a poor prognosis and resistance to DNA-damaging chemotherapeutic agents. This association does not apply to BRCA1 cancers, in which repair by HR between sister chromatids may be compromised by BRCA1 deficiency. RAD21 is thus a potential BRCA1/2 mutation status-dependent predictive and prognostic marker in familial breast cancers.

## Abbreviations

BRCA1: breast cancer 1, early onset; BRCA2: breast cancer 2, early onset; CGH: chromogenic hybridization; CISH: chromogenic *in situ *hybridization; CNV: copy-number variation; DCIS: ductal carcinoma *in situ*; DSB: double-stranded break; ER: estrogen receptor; HER2: human epidermal growth receptor 2; HR: homologous recombination; miRNA: microRNA; PARP: poly-ADP ribose polymerase; SNP: single-nucleotide polymorphism.

## Competing interests

The authors declare that they have no competing interests.

## Authors' contributions

MY contributed to the conception and design of the study, analyzed the data, and drafted and revised the manuscript. HX contributed to the conception and design of the study, performed the immunohistochemistry, and revised the manuscript. NW performed the CGH and mRNA array experiments. KS performed the miRNA experiments. IH contributed to the statistical analysis and design of the miRNA array experiments. kConFab contributed to the provision of study materials and the collection of clinicopathologic and follow-up survival data. MJM contributed to the conception and design of the study and the analysis, drafting, and revision of the manuscript. SBF contributed to the conception and design of the study, provision of study materials, analysis, drafting, and revision of the manuscript. All authors read and approved the final manuscript.

## Supplementary Material

Additional file 1 **Table S1**. Flow of familial breast cancer patients through the study, according to REMARK criteria. Click here for file

Additional file 2 **Table S2**. Nuclear RAD21 expression in familial breast cancers (score out of 7). Click here for file
