# Supplementary material for: Enhanced RAD21 cohesin expression confers poor prognosis in BRCA2 and BRCAX, but not BRCA1 familial breast cancers
Source: Breast Cancer Res. 2012 Apr 26;14(2):R69. doi: 10.1186/bcr3176 (PMC3446404; doi:10.1186/bcr3176)
Supplement: Additional file 1 — Table S1. Flow of familial breast cancer patients through the study, according to REMARK criteria. [file bcr3176-S1.DOC]

**Table S1:** Flow of familial breast cancer patients through the study, according to REMARK criteria[16].

|  | **BRCA1** | **BRCA2** | **BRCAX** | **Total** |
| --- | --- | --- | --- | --- |
| 1. Female patients collected for study | 45 | 36 | 66 | 147 |
| 1. Patients with tissue available | 42 | 34 | 63 | 139 |
| 1. Tumour present on microarray | 33 | 32 | 56 | 121 |
| 1. Tumours with staining for RAD21 and intrinsic subtypes plus survival data | 28 | 27 | 39 | 94 |
