# Supplementary material for: Enhanced RAD21 cohesin expression confers poor prognosis in BRCA2 and BRCAX, but not BRCA1 familial breast cancers
Source: Breast Cancer Res. 2012 Apr 26;14(2):R69. doi: 10.1186/bcr3176 (PMC3446404; doi:10.1186/bcr3176)
Supplement: Additional file 2 — Table S2. Nuclear RAD21 expression in familial breast cancers (score out of 7). [file bcr3176-S2.DOC]

**Table S2a.** Nuclear RAD21 expression in familial breast cancers (score out of 7)

| **Score** | **BRCA1** | **BRCA2** | **BRCAX** |
| --- | --- | --- | --- |
| **0** | 6 (21%) | 4 (15%) | 11 (28%) |
| **2** | 0 | 0 | 0 |
| **3** | 1 (4%) | 2 (8%) | 2 (5%) |
| **4** | 5 (18%) | 4 (15%) | 4 (10%) |
| **5** | 5(18%) | 5 (18%) | 9 (23%) |
| **6** | 3 (11%) | 5 (18%) | 4 (11%) |
| **7** | 8 (28%) | 7 (26%) | 9 (23%) |

**Table S2b.** Nuclear RAD21 expression among the intrinsic subtypes in familial breast cancers (score out of 7)

| **Score** | **Luminal** | **Basal** | **HER2** | **Null** |
| --- | --- | --- | --- | --- |
| **0** | 8 (19%) | 7(22%) | 1 (25%) | 2 (40%) |
| **2** | 0 | 0 | 0 | 0 |
| **3** | 0 | 3 (9%) | 0 | 0 |
| **4** | 4 (10%) | 5 (16%) | 0 | 2 (40%) |
| **5** | 12 (29%) | 4 (12%) | 1 (25%) | 0 |
| **6** | 4 (10%) | 5 (16%) | 0 | 1 (20%) |
| **7** | 13 (32%) | 8 (25%) | 2 (50%) | 0 |
